# Agreement Between High-Resolution Ultrasound and Electro-Physiological Examinations for Diagnosis of Carpal Tunnel Syndrome in the Indonesian Population

**DOI:** 10.3389/fneur.2019.00888

**Published:** 2019-08-26

**Authors:** Dessy R. Emril, Iskandar Zakaria, Mirza Amrya

**Affiliations:** ^1^Department of Neurology, Faculty of Medicine, Universitas Syiah Kuala, Dr. Zainoel Abidin General Hospital, Banda Aceh, Indonesia; ^2^Department of Radiology, Faculty of Medicine, Universitas Syiah Kuala, Dr. Zainoel Abidin General Hospital, Banda Aceh, Indonesia

**Keywords:** carpal tunnel syndrome, ultrasonography, sensitivity, specificity, electrodiagnostic, kappa value

## Abstract

Carpal tunnel syndrome (CTS) is a disorder of the wrist due to narrowing of the carpal tunnel. It can be caused by trauma or tumors in the tunnel resulting in compression of the median nerve. This disorder is often diagnosed with early symptoms such as tingling, numbness, and weakness that subsequently lead to hand muscle atrophy. While ultrasonography (USG) is one of the diagnostic methods of CTS, neurophysiological diagnosis, such as with nerve conduction study (NCS), is standard in clinics where the necessary equipment is available. This cross-sectional study aimed to compare USG diagnostic values with NCS results to determine USG efficacy for diagnosis of CTS. Data on medical history, physical examination, ultrasound results, and NCS examination from patients who had been diagnosed with CTS at a regional general hospital in Indonesia were collected. In total, 46 patients participated in the study and data were compared using 2 × 2 table analyses and the kappa statistic. Results showed USG sensitivity, specificity, positive predictive value, negative predictive value, positive likelihood ratio, negative likelihood ratio, and accuracy values of 88.5, 65, 76.6, 81.25, 2.52, 0.17, and 78.2%, respectively (*p* < 0.005). Comparison between NCS and the USG assessment obtained a kappa coefficient of κ = 0.71 and showed high agreement (κ = 0.410.60). In conclusion, the diagnostic value of USG compared to NCS is acceptable. Therefore, USG examination is a feasible CTS diagnostic alternative for clinicians who do not have access to an electrodiagnostic facility.

## Introduction

Carpal tunnel syndrome (CTS) results in a complex set of symptoms due to entrapment of the median nerve by the transverse carpal ligament within the carpal tunnel of the wrist (1). Initially, symptoms include sensory disorders in the form of paresthesia, numbness, or tingling of the fingers (2). Motoric symptoms occur in severe conditions, which can lead to atrophy of the thenar muscle or other muscles innervated by the median nerve and can have a negative impact on quality of life (2, 3). According to the labor statistics agency report, CTS is a disease that often occurs among industrial workers in developed countries. Based on a study conducted by the American Academy of Orthopedic Surgeons in the United States, the incidence of CTS is 1–3 cases per 1,000 people per year, with a prevalence of 50 cases per 1,000 people in the general population. However, the incidence can be as high as 150 cases per 1,000 people per year, with a prevalence of 500 per 1,000 people in high-risk groups. The ratio of women to men with CTS is 3–10: 1 (4, 5) and CTS is most prevalent in the 45–60 y age group (6).

In addition to clinical symptoms, the diagnosis of CTS can be strengthened by physical, neurophysiological, radiological, and laboratory examination (2). Neurophysiological analysis includes electromyography (EMG) nerve conduction study (NCS). According to Aroori, EMG-NCS examination is the standard in diagnosing CTS with sensitivity between 49 and 84% and specificity between 95 and 99% (7). However, the introduction of needles into the muscle during the examination is invasive and can be painful, causing some patients to be reluctant (8). In addition, the equipment for the EMG-NCS is relatively expensive and can only be found in certain health service centers.

Another diagnostic method of CTS is ultrasonography (USG) examination. USG allows for visualization of anatomy and nerve form directly with fairly good sensitivity and specificity values of 77.6 and 86.8%, respectively (9). Examination using USG is more comfortable for patients because the transducer is only in light contact with the skin of the patient's hand (7, 9). Further, in Indonesia, not all hospitals and health service centers have EMG-NCS equipment, but USG facilities are common.

There has been a lack of studies focused on the efficacy of USG for CTS diagnosis in the Indonesian population. This study aims to find the diagnostic value of USG for CTS in a sample of Indonesian patients by comparing diagnostic results from USG with those from EMG-NCS.

## Research Methodology

### Patient Selection

This was a diagnostic study with an analytic cross-sectional approach conducted from October 1 to December 30, 2017. The population were, all patients diagnosed with CTS at the Neurology Polyclinic located at the Regional General Hospital Dr. Zainoel Abidin in Banda Aceh, Indonesia. Inclusion criteria were patients diagnosed with CTS according to clinical findings of CTS, willingness to participate in the study, and able to follow the entire series of examinations. Exclusion criteria were patients who refused to take part in the examination, pregnant women, and postoperative CTS patients. This study was carried out in accordance with the recommendations of CIOMS 2016 guidelines, Ethical Committee of Medical Faculty, Syiah Kuala University number 66/KE/FK/2017. The protocol was approved by the Ethical Committee of Medical Faculty, Syiah Kuala University. All subjects gave written informed consent in accordance with the Declaration of Helsinki.

### Nerve Conduction Study

All patients who were suspected of having CTS underwent standard median distal motor nerve latency (DML) studies (Technique H) by a neurologist with a protocol based on a summary statement from the American Academy of Neurology regarding CTS diagnosis (10). Motor median conduction studies were performed using standard supramaximal stimulation techniques. The DML was measured by an active electrode placed above the muscle belly of the M. abductor pollicis brevis. The nerve was stimulated using bipolar stimulation electrodes with the cathode positioned 2 cm proximal to the wrist fold. The cathode was placed closest to the recording electrode. CTS was positive if the NCS showed prolonged DML (>4.0 ms) (11).

### Ultrasonography Scanning Method and Diagnostic Protocol

**Figure 1:**
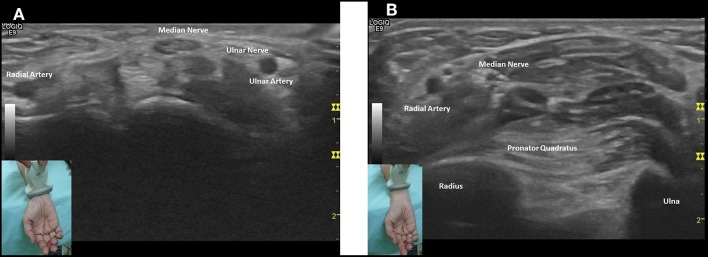
Transverse images of the median nerve with cross-sectional area measurements at two levels: **(A)** CSAc at the wrist fold and **(B)** CSAp at the pronator quadratus muscle.

The radiologist who performed median nerve USG in the forearm was blinded to clinical and NCS data. The examination of the median nerve was performed using a 5–12 MHz linear-array transducer of GE® logic 9 US machine. The USG settings such as frequency, depth, and focal zone, were optimized for nerve imaging. The probe was maintained at a perpendicular angle during analysis to prevent anisotropy and median nerve deformation, and additional weight was not permitted when pressure was applied to the skin surface with the probe. Subjects were seated facing the examiner. With extended arms, the wrists rested on a hard surface, with forearms supinated and fingers half-extended. Transverse images f the median nerve with cross-sectional area measurements were obtained at two levels; at the CSAc at the wrist fold (CSAc = carpal tunnel cross-sectional area) and the pronator quadratus level (CSAp = proximal cross-sectional area) (Figure 1) (12). Result of measurement both arms were examined using the same technique. The value of the difference between CSAc and CSAp (Δ CSA) was calculated for each wrist.

Measuring of the CSA of the median nerve was done by tracing method. The subject was diagnosed has carpal tunnel syndrome if the difference between CSAc and CSAp (ΔCSA) ≥2 mm^2^.

### Statistical Analysis

The sampling technique used a diagnostic test sample formula with a sample size of 46 samples. The USG and NCS results were compared using 2 × 2 tables and analyzed by the Receiver Operating Characteristic (ROC) curve. Next, the reliability was compared using the kappa statistic.

## Results

The frequency distribution of participant characteristics are presented in Table 1.

**Table 1:** Frequency distribution of participant characteristics.

**Variable**	**Frequency**	**Percentage (%)**
**Gender**		
Male	9	19.6
Female	37	80.4
**Age (years)**		
<40	7	15.2
40–60	31	67.4
>60	8	17.4
**Location of CTS**		
Left	24	52.2
Right	22	47.8
**Diabetes Mellitus**		
Yes	0	0
No	46	100
**Hypertension**		
Yes	19	41.3
No	27	58.7
**Durations of onset of CTS**		
<1 year	25	54.3
≥1 year	21	45.7

*CTS, carpal tunnel syndrome*.

Table 1 shows that CTS was diagnosed more frequently in women (80.4%) than in men (19.6%) and individuals 40–60 years old had the highest prevalence of CTS (67.4%). The affected hand was similar between the left side (52.2%) and right side (47.8%). There were 41.3% of participants with hypertension and no patients with a history of diabetes mellitus. There was almost no difference in duration of onset of CTS from <1 year and ≥1 year.

### Ultrasonography and Electrodiagnostic Results

Table 2 shows the mean tunnel inlet (CSAc) measured by USG was 14.87 mm^2^, with 12.39 mm^2^ for the mean of the pronator quadratus muscle (CSAp) inspection, and 3.06 mm^2^ for the mean change (Δ CSAc-CSAp). The mean DML was 4.96 ms ± 1.721.

**Table 2:** Ultrasonography and electrodiagnostic results.

	**Parameters**	**Mean**	**Standard Deviation (SD)**
Ultrasonography (mm^2^)	Tunnel Inlet (CSAc)	14.87	2.254
	Pronator quadratus (CSAp)	12.39	1.638
	Δ CSAc-CSAp	3.06	2.100
Electrodiagnostic (ms)	Median Distal Motor Latency	4.96	1.721

### Comparison Analysis Based on Ultrasonography Results

The statistical analysis presented in Table 3, shows that there is a significant difference between positive (15.74 mm^2^) and negative (13.26 mm^2^) CTS patients in tunnel inlet (CSAc) mean (*p* < 0.001). In addition, the mean Δ CSAc-CSAp also shows a significant difference between positive (4.23 mm^2^) and negative (0.88 mm^2^) CTS patients (*p* < 0.001).

**Table 3:** Ultrasonography result value.

	**Mean CSAc (mm^**2**^)**	***P*-value**	**Mean CSAp (mm^**2**^)**	***P*-Value**	**ΔCSAc-CSAp (mm^**2**^)**	***P*-value**
Positive	15.74		11.89		4.23	
		0.000		0.007		0.000
Negative	13.26		13.32		0.88	

### Comparison of Ultrasonography and Electrodiagnostic Results

**Figure 2:**
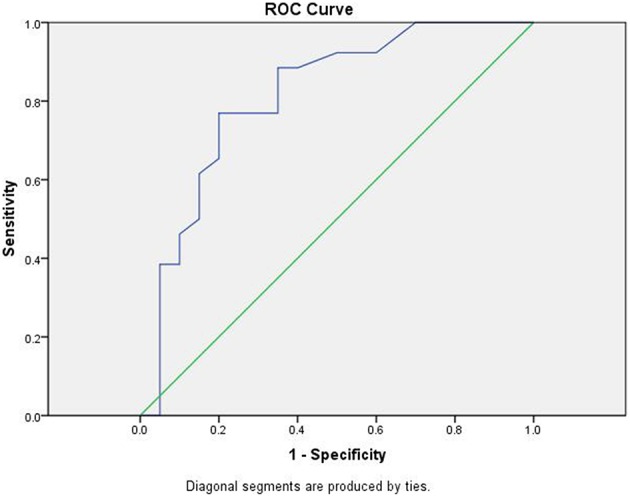
ROC Curve of ultrasonography results.

In this study, the results of ultrasound examinations were performed on patients with suspected CTS. The interpretation of the results was grouped by diagnosis into CTS and not CTS. Next, electrodiagnostic results were used to classify patients into CTS and not CTS groups. The resulting groups were analyzed to determine validity of the diagnostic value for USG and are presented in Table 4. USG results showed a sensitivity value of 88.5%, specificity of 65%, positive predictive value of 76.6%, negative predictive value of 81.25%, positive likelihood ratio of 2.52, negative likelihood ratio of 0.17, and accuracy of 78.2%. The results of the ROC curve analysis of ultrasound examination with electrodiagnostic can be seen in Figure 2.

**Table 4:** Comparison of ultrasonography and electrodiagnostic results.

		**Positive**	**Negative**	**Total**
**ELECTRODIAGNOSTIC RESULT**				
USG Result	Positive	23	7	30
	Negative	3	13	16
	Total	26	20	46

### Ultrasound Results Based on ROC Curve Analysis

The ROC curve analysis shows the ultrasound examination curve above the 50% line, approaching toward 100%. The area under the curve (AUC) value obtained from the ROC method was 81.3% (95% CI: 68.2–94.5, *p* < 0.001). Statistically, the AUC value of 81.3 is good. An AUC value of 81.3% with Δ CSAc-CSAp cut-off value of 2.10 mm^2^ means that if ultrasound examination is used to diagnose the possibility of CTS in 46 patients, then the right conclusion will be obtained in 37 patients. This study also carried out a comparative analysis between the results of ultrasound examination with NCS for agreement in diagnosing CTS. The comparison between the two examinations is presented in Table 5.

**Table 5:** Table Kappa 2 × 2.

		**Electrodiagnostic**		
		**Negative**	**Positive**	**Total**
USG Result	Negative	13	3	16
	Positive	7	23	30
	Total	20	26	46

### Kappa Analysis Between Ultrasonography and Electrodiagnostic

From Table 5, the results of the test using kappa statistic is 0.71, which indicates that there is a good match between USG and NCS examination results.

## Discussion

This cross-sectional study aimed to compare USG with NCS diagnostic results for CTS to determine USG efficacy in settings without access to NCS. The results of comprehensive statistical analysis show that in terms of sensitivity, specificity, positive predictive value, negative predictive value, positive likelihood ratio, negative likelihood ratio, and accuracy, USG examination has effective diagnostic value when compared to NCS.

When the peripheral nerve is entrapped, US imaging can reveal nerve flattening at the compressed site and swollen nerve fascicles, which are proximal to the level of compression (13). Changes in the nerve's shape and size are common sonographic findings of entrapment neuropathy and measurement of the nerve's cross-sectional area is the most commonly employed indicator to differentiated between normality and pathology (14). According to Fowler et al., various results of research examining USG diagnostic testing in CTS patients have varied sensitivity results, ranging from 57 to 98%, while the specificity varies between 51 to 100% (9). Yazdchi study et al. obtained results of sensitivity between 70 to 89% and specificity of 57 to 97% (15). The ROC curve also shows that the values found are 81.3%, so it can be interpreted that ultrasound has good diagnostic value in comparison with electrodiagnostic study in CTS patients. In this study, the cut-off value for Δ CSAc-CSAp was 2.10 mm^2^. This result was consistent with previous studies that mention a Δ CSAc-CSAp threshold of 2 mm^2^ to yield the greatest sensitivity (99%) and specificity (100%) for the diagnosis of CTS. Based on these results, USG is proven to be useful as an alternative tool in diagnosing CTS (12).

This study also compared the strength of agreement between USG examination and NCS in establishing a CTS diagnosis. The results of the analysis using the kappa statistic to compare the inter-rater agreement had a good level of agreement. In their study Shahebari et al. obtained a kappa value of 0.71 to 0.78 (good) in assessing the accuracy of USG in establishing a diagnosis of CTS using a median nerve area (16). In another study, Lee et al. only obtained a kappa coefficient of 0.55 (moderate) in assessing bone size in diagnosing CTS using ultrasound (17). This study concludes that this examination has a good strength of agreement and USG and NCS examination can be considered comparable diagnostic options for CTS.

The results showed that there were fewer men with CTS than women. Of the 46 people with CTS clinical signs, there were 37 women (80.4%) and 9 men (19.6%) with a ratio of 4:1. This result is similar to the majority of the literature, which states that women are more often affected by CTS than men, but with a ratio of 1.4:1 (18). Parviizi and Kim report that CTS is also more common in women but with a ratio 3–10:1 (4). According to Duncan et al., this is likely because women have a smaller wrist size than men which causes the size of the carpal tunnel to become smaller in general. In addition, women also experience hormonal changes that affect changes in the tenosynovial tissue in the carpal tunnel (19). Age distribution ranged from 24 to 63 years. Based on the present cases, most were found in the age groups 46–60 and 31–45 years. This is in accordance with the literature which states that CTS is often diagnosed in patients aged 45–60 years and only 10% of patients younger than 31 years are diagnosed with CTS (4, 20). Based on the distribution of clinical findings, 24 people (52.2%) experienced symptoms in the left hand and 22 people (47.8%) in the right hand, which aligns with the literature that states CTS often occurs in the right hand (4, 20).

Based on the distribution, according to concomitant diseases, 19 (41.3%) patients had a history of hypertension, while 27 (58.7%) other patients had no history of hypertension. Based on the literature, hypertension and diabetes mellitus are the most common risk factors for CTS disease, but in this study, there were no patients who had a history of diabetes mellitus (21, 22). Based on the distribution according to the duration of suffering from CTS, 25 (54.3%) had been diagnosed with CTS for <1 year, while 21 (45.7%) patients were diagnosed with CTS for more than 1 year.

USG examination can be used in centers that do not have NCS as a standard CTS diagnostic tool (17). High-resolution USG enables clear visualization of nerve form and texture. The use of ultrasound in a clinic setting offers benefits of no radiation, portability, and excellence in visualization of muscles and surrounding connective tissue (23).

## Conclusion

The results of this study showed that the USG examination had excellent level of agreement in diagnosing CTS compared to NCS. For this reason, ultrasound examination can be used as an additional or alternative diagnostic tool in diagnosing CTS patients. The recommendations for further research are to link the results of USG with NCS toward motoric and sensory quality in CTS patients. For clinicians, the researchers suggest that this study can be used as a reference in establishing a diagnosis of CTS in centers without an electrodiagnostic facility.

## Data Availability

All datasets generated for this study are included in the manuscript and/or the supplementary files.

## Ethics Statement

This study was carried out in accordance with the recommendations of CIOMS 2016 guidelines, Ethical Committee of Medical Faculty, Syiah Kuala University number 66/KE/FK/2017. The protocol was approved by the Ethical Committee of Medical Faculty, Syiah Kuala University. All subjects gave written informed consent in accordance with the Declaration of Helsinki.

## Author Contributions

DE substantially contributed to the conception and design of the work, as well as the acquisition, analysis, and interpretation of data for the work. IZ had a substantial contribution in the ultrasonography examination and design of the work. MA substantially contributed to the collection and analysis of data.

### Conflict of Interest Statement

The authors declare that the research was conducted in the absence of any commercial or financial relationships that could be construed as a potential conflict of interest.
